# Intravenous Dexmedetomidine Use in Total Hip Arthroplasty May Lead to Elevated Rates of Hypotension

**DOI:** 10.7759/cureus.43768

**Published:** 2023-08-19

**Authors:** Christopher T Holland, Andrew J Meyers, Zachary C Lum, Hailee Tougas, Mauro Giordani, John P Meehan

**Affiliations:** 1 Orthopaedic Surgery, Duke University Medical Center, Durham, USA; 2 Orthopaedics, University of California (UC) Davis School of Medicine, Sacramento, USA; 3 Orthopaedic Surgery, University of California (UC) Davis School of Medicine, Sacramento, USA; 4 Orthopaedic Surgery, Nova Southeastern University, Pembroke Pines, USA; 5 Orthopaedics, University of California (UC_ Davis School of Medicine, Sacramento, USA

**Keywords:** ambulatory arthroplasty, hypotension, enhanced recovery after surgery, total hip arthroplasty, intravenous dexmedetomidine

## Abstract

Purpose

Enhanced recovery protocols for total hip arthroplasty (THA), using opioid-sparing techniques have become widely used. Reports of novel additions to multimodal pain control regimens have been published, however, a paucity of literature exists on the use of intravenous dexmedetomidine. In this study, we analyzed our experience with intravenous dexmedetomidine and hypothesized that it would reduce postoperative opioid use. Secondary outcomes were also examined, including post-operative hypotension, hemoglobin, length of stay, and discharge disposition.

Methods

All patients who underwent primary THA at a single tertiary-level center between January 1, 2016, and September 1, 2019, underwent investigation. Diagnosis, surgical approach, anesthetic type, body mass index (BMI), and American Society of Anesthesiologists (ASA) score were recorded. Postoperative clinical measures were analyzed, adjusting for patient and surgical characteristics.

Results

Of the 599 patients included in the study, 218 patients received intravenous (IV) dexmedetomidine, at a mean dose of 44.9 mg during their operative event. Using a multivariate model, patients in the IV dexmedetomidine group were estimated to have received 24% elevated morphine milligram equivalent at postoperative day zero compared to those in the control group (p = 0.05). In addition, patients in the IV dexmedetomidine group who underwent spinal anesthesia had increased odds of hypotension 3.47 times that of the control [odds ratio (OR) 1.43-8.43, p=0.006].

Conclusions

Surprisingly, we found no opioid-sparing effects with the use of IV dexmedetomidine. IV dexmedetomidine may be used cautiously as an anesthesia adjunct with spinal anesthesia in the setting of primary THA, as the experience at our institution illustrated increased odds of postoperative hypotension.

Level of evidence

This retrospective case-control study has a level of evidence III.

## Introduction

Total hip arthroplasty (THA) is a common elective procedure performed in the United States, with over 370,000 primary THAs completed in 2014 and more than 630,000 procedures projected in the year 2030 [1]. With multimodal analgesia and rapid recovery shortening the length of hospitalization, an increased emphasis has been placed on decreasing opioid utilization for perioperative analgesia [2].

While many institutions have now implemented multimodal pain control regimens in conjunction with Enhanced Recovery After Surgery Protocols (ERAS) to manage perioperative pain in THA patients, there is wide variation in the interpretation and implementation of such protocols. Generally, a multimodal pain regimen includes cryotherapy, acetaminophen, non-steroidal anti-inflammatory drugs (NSAIDs), gabapentinoids, and opioids, with the concurrent use of peripheral nerve blockade and/or periarticular injections [3-4]. More recently, reports of novel additions to these regimens have been published. In 2018, one institution reported their experience with the use of intraoperative intranasal (IN) dexmedetomidine in primary total hip arthroplasty patients who underwent general anesthesia in Finland [5]. Dexmedetomidine is a highly selective a2-adrenoceptor agonist that can produce sedative, anxiolytic, and sympatholytic results while minimizing many of the undesirable physiologic effects of more traditional medications used for similar applications [6]. In their study, they reported the use of a low dose (50 mg) intranasal (IN) dexmedetomidine was able to demonstrate a clinically significant reduction in postoperative opioid consumption [5]. This finding was promising, however, a paucity of literature exists on the use of intravenous (IV) dexmedetomidine in a broader patient population.

Dexmedetomidine was approved in 2003 for procedural sedation by the Food and Drug Administration. Pharmacokinetic studies have demonstrated the hepatic metabolism of dexmedetomidine with a terminal elimination half-life of approximately two hours, with the pharmacodynamic properties of a potent and highly selective a2-adrenoceptor agonist exhibiting sedative, anxiolytic, sympatholytic, and analgesic-sparing effects, while having a minimal impact on respiratory depression [6-7]. The sedative and anxiolytic effect has widened the application of dexmedetomidine to be used as a common adjuvant to anesthesia techniques.

We report our institutional experience with the intraoperative use of IV dexmedetomidine in 218 total hip arthroplasty patients and compare their inpatient opioid consumption and postoperative clinical measures with 381 patients over a similar timespan who did not receive IV dexmedetomidine. A retrospective evaluation was performed on this patient population to determine the impact of intraoperative IV dexmedetomidine use on perioperative opioid consumption, postoperative day (POD) zero hypotension, postoperative day one hemoglobin, length of stay (LOS), and discharge disposition. Our hypothesis was that IV dexmedetomidine use would decrease perioperative opioid consumption.

## Materials and methods

After approval by the University of California: Davis Institutional Review Board, IRB #1355396-1, patients who underwent primary THA at a single academic quaternary care center between January 1, 2016, and September 1, 2019, were retrospectively identified. To minimize selection bias, all patients greater than 18 years of age, regardless of underlying diagnosis, surgical approach, anesthetic type, body mass index (BMI), or American Society of Anesthesiologists (ASA) score, were included. Six hundred and four subjects, representing 604 separate operative events by primarily three adult reconstruction fellowship-trained orthopedic surgeons, were identified during this time period. Patients were excluded from the study if they were less than 18 at the time of surgery and five patients with a length of stay greater than seven days were removed from the data set, to address gross outliers, leaving 599 subjects for final review. Anesthetic type, general versus neuraxial, was determined by the anesthesiologist, as was the administration of peripheral nerve blockade. Intraoperative IV dexmedetomidine administration was provided at the discretion of the anesthesia provider without knowledge of, or recommendation for or against, by the surgeon. In this study cohort, the use of the intraoperative administration of IV dexmedetomidine, the dose, and administration was determined by each anesthesiologist. The mean dose of IV dexmedetomidine delivered was 44.9mg (range 4 - 1034 mg). A standardized multimodal pain management protocol was utilized for all patients consisting of routine use of a preoperative non-steroidal anti-inflammatory (celecoxib), gabapentinoid, and acetaminophen. In addition, all patients receive weight-based prophylactic intravenous antibiotic dosing prior to incision, a single one-gram loading dose of tranexamic acid (TXA) at the time of sterile draping, and a single dose of 10 mg intravenous dexamethasone. Postoperatively all patients are prescribed oral oxycodone every four hours as needed: a 5 mg dose is given for moderate pain or a 10 mg dose is given for severe pain. A single additional rescue dose of oxycodone 5 mg is given if the pain is not relieved after one hour. Moderate pain is defined as 4-6 on the visual analog scale (VAS) pain score, severe pain is defined as 7-10 on VAS pain score.

Postoperative hypotension, hemoglobin, daily opioid consumption, length of stay, and discharge disposition were then analyzed, adjusting for patient and surgical characteristics. Hypotension was defined as mean arterial pressure less than 60, using routine nursing data from blood pressure cuff measurements.

Postoperative opioid doses, in morphine milligram equivalents (MME), were compared between the group that received IV dexmedetomidine and the group that did not receive IV dexmedetomidine at each postoperative day, adjusting for patient and surgical characteristics including baseline opioid use, using a linear mixed-effects model.

With the known sympatholytic effects of IV dexmedetomidine, we were also interested in characterizing the rates of hypotension at POD 0. The hypotensive events and rates of discharge to a skilled nursing facility were compared between the group that received IV dexmedetomidine and the group that did not receive IV dexmedetomidine using mixed effects logistic regression models.

Continuous patient and surgical characteristics were compared between the group that received IV dexmedetomidine and the group that did not receive IV dexmedetomidine using Wilcoxon rank-sum tests. Categorical patient and surgical characteristics were compared between the group that received IV dexmedetomidine and the group that did not receive IV dexmedetomidine using Fisher’s Exact test. The linear mixed effects models included random intercepts for subject and surgeon and fixed effects for dexmedetomidine use, age, sex, BMI, ASA score, type or use of regional nerve block, use of pericapsular injection, type of anesthesia, approach, opioid use at baseline, day, and the interaction of day with all other variables. Postoperative MME doses were log transformed prior to analysis in order to more closely satisfy model assumptions.

Analyses were conducted using R version 4.0.0 Patched (2020-05-18 r78487). The linear mixed effects model was fitted using the R package nlme, version 3.1-148, and the mixed effects logistic regression models were fitted using the R package lme4, version 1.1-23. Post-hoc power analysis confirmed 98.9% power to detect differences for opioid use.

## Results

Of the 599 patients included in the study, 218 patients received IV dexmedetomidine, at a mean dose of 44.9 mg. Table 1 shows patient and surgical characteristics by dexmedetomidine use with no significant difference between patient age, postoperative day one hemoglobin, sex, ASA status, the administration of pericapsular injection, operative laterality, and length of hospital stay, in the cohort who received IV dexmedetomidine and the cohort who did not. Patients who received IV dexmedetomidine had a significantly higher mean BMI (p = 0.01), were significantly more likely to have received general anesthesia (p < 0.001), to have had an anterior approach used in surgery (p = 0.003), to have received a regional nerve block (p = 0.029), to have received preoperative decadron (p < 0.001), to have received intraoperative opioids (p < 0.001), to use opioids at baseline (p = 0.012), and to use gabapentinoids at baseline (p = 0.013).

**Table 1:** Continuous variables were compared between groups using Wilcoxon rank-sum tests and categorical variables were compared between groups using Fisher’s exact test. Patients who received dexmedetomidine had a significantly higher (but perhaps not clinically meaningfully higher) mean BMI than patients who did not receive dexmedetomidine (p = 0.01). Patients who received dexmedetomidine were significantly more likely to have received general anesthesia (p = <0.001), to have had an anterior approach used in surgery (p = 0.0033), to have received a regional nerve block (p = 0.029), to have received preoperative decadron (p = 0.0018), to have received intraoperative opioids (p = 0.0018), to use opioids at baseline (p = 0.012), and to use gabapentinoids at baseline (p = 0.013). Length of stay (LOS).

	No Dex (n=381)	Dex Used (IV) (n=218)	All Patients (n=599)	P value
DEX dose (ug)				<0.001
N	381	218	599	
Mean (SD)	0 (0)	44.9 (74.4)	16.3 (49.8)	
Median (Range)	0 (0-0)	34.5 (4-1034)	0 (0-1034)	
Age				0.5236
N	381	218	599	
Mean (SD)	64.1 (11.2)	63.3 (11.6)	63.8 (11.3)	
Median (Range)	64 (22-93)	64 (21-89)	64 (21-93)	
BMI (kg/m2)				0.0105
N	381	218	599	
Mean (SD)	28.5 (5)	29.8 (5.7)	29 (5.3)	
Median (Range)	28.5 (18.3-43.6)	29.2 (15.1-49.5)	28.6 (15.1-49.5)	
POD 1 Hemoglobin				0.7937
N	369	213	582	
Mean (SD)	11.3 (5)	11.1 (1.5)	11.2 (4.1)	
Median	11.1	11	11.1	
Sex				0.1732
F	212 (55.6%)	108 (49.5%)	320 (53.4%)	
M	169 (44.4%)	110 (50.5%)	279 (46.6%)	
ASA Score				0.6733
1	15 (3.9%)	7 (3.2%)	22 (3.7%)	
2	212 (55.6%)	119 (54.6%)	331 (55.3%)	
3	151 (39.6%)	92 (42.2%)	243 (40.6%)	
4	3 (0.8%)	0	3 (0.5%)	
Anesthetic Type				<0.001
General	150 (39.4%)	139 (63.8%)	289 (48.2%)	
General, Spinal	13 (3.4%)	2 (0.9%)	15 (2.5%)	
Spinal	214 (56.2%)	77 (35.3%)	291 (48.6%)	
Operative Side				0.2918
BL	1 (0.3%)	1 (0.5%)	2 (0.3%)	
L	175 (45.9%)	87 (39.9%)	262 (43.7%)	
R	205 (53.8%)	130 (59.6%)	335 (55.9%)	
Approach				0.0033
Anterior	19 (5%)	26 (11.9%)	45 (7.5%)	
Posterior	362 (95%)	192 (88.1%)	554 (92.5%)	
Regional Nerve Block				0.0293
No	225 (59.1%)	109 (50%)	334 (55.8%)	
Yes - fascia iliaca	132 (34.6%)	84 (38.5%)	216 (36.1%)	
Yes - femoral nerve	0	1 (0.5%)	1 (0.2%)	
Yes - quadratus lumborum	24 (6.3%)	24 (11%)	48 (8%)	
Preoperative Decadron				0.0018
No	280 (73.5%)	133 (61%)	413 (68.9%)	
Yes	101 (26.5%)	85 (39%)	186 (31.1%)	
Preoperative NSAID				0.8291
No	73 (19.2%)	40 (18.3%)	113 (18.9%)	
Yes	308 (80.8%)	178 (81.7%)	486 (81.1%)	
Preoperative NSAID (celebrex, mg)				0.1488
N	381	218	599	
Mean (SD)	237 (146.8)	221.1 (136.2)	231.2 (143.1)	
Median (Range)	200 (0–400)	200 (0–400)	200 (0–400)	
Preoperative Gabapentin				0.1042
No	44 (11.5%)	36 (16.5%)	80 (13.4%)	
Yes	337 (88.5%)	182 (83.5%)	519 (86.6%)	
Preoperative Gabapentin (mg)				0.0170
N	381	218	599	
Mean (SD)	307.8 (190.4)	269.8 (167)	294 (183)	
Median (Range)	300 (0–900)	300 (0–900)	300 (0–900)	
Intraoperative Opioid				0.0018
No	114 (29.9%)	40 (18.3%)	154 (25.7%)	
Yes	267 (70.1%)	178 (81.7%)	445 (74.3%)	
Intraoperative Opioid (MME)				<0.001
N	381	218	599	
Mean (SD)	14.6 (24)	21.8 (17.1)	17.2 (22)	
Median (Range)	10 (0–400)	25 (0–89)	15 (0–400)	
Pericapsular Injection				0.7973
No	163 (42.8%)	96 (44%)	259 (43.2%)	
Yes	218 (57.2%)	122 (56%)	340 (56.8%)	
Opioid Use at Baseline				0.0120
No	242 (63.5%)	115 (52.8%)	357 (59.6%)	
Yes	139 (36.5%)	103 (47.2%)	242 (40.4%)	
Gabapentinoid Use at Baseline				0.0126
No	328 (86.1%)	170 (78%)	498 (83.1%)	
Yes	53 (13.9%)	48 (22%)	101 (16.9%)	
LOS				0.6105
N	381	218	599	
Mean (SD)	3.6 (1)	3.5 (1.1)	3.6 (1)	
Median (Range)	3 (2–7)	3 (2–7)	3 (2–7)	

**Figure 1:**
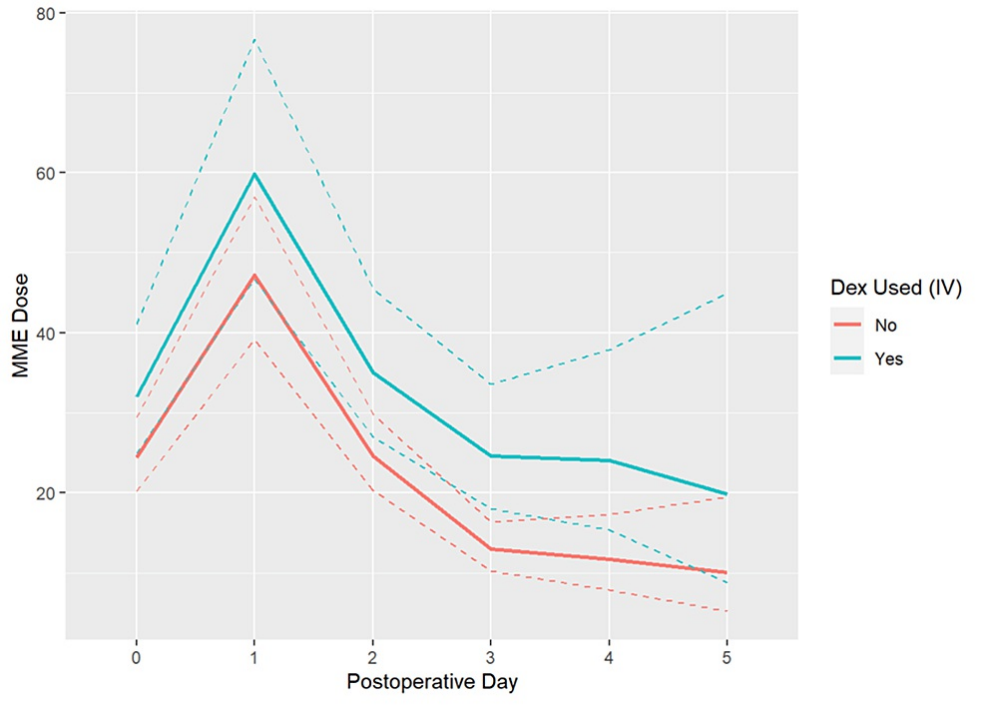
Estimates from a linear mixed effects model (without covariate adjustment) of MME doses by group and timepoint. Solid lines show model estimates and dashed lines show 95% confidence intervals. Patients who received IV dexmedetomidine are estimated to have a post-operative day zero MME 24% higher than those in the control group (p = 0.05). Post-operative opioid consumption in the IV dexmedetomidine group showed a trend towards increased MME and was estimated to be 11, 9, and 30% higher, compared to the group that did not receive IV dexmedetomidine, on postoperative days 1, 2, and 3.

Figure 1 demonstrates results from the linear mixed effects model highlighting the effect of IV dexmedetomidine along with other patient and surgical characteristics on MME dose at postoperative days zero through three. After adjusting for all other variables in the model, including baseline opioid use, patients in the dexmedetomidine group had a post-operative day zero MME 24% higher than those in the control group (p = 0.05). Opioid consumption on POD 1-3 in the IV dexmedetomidine group did not have a significant reduction in MME and was actually estimated to be up to 30% higher, compared to the group that did not receive IV dexmedetomidine, however, this did not reach significance.

Postoperative hypotension was evaluated between the group that received IV dexmedetomidine and the group that did not. Based on a multivariate linear mixed effects model, the effect that IV dexmedetomidine and other patient and surgical characteristics had on hypotension at POD 0 is listed in Table 2. Adjusting for all other variables in the model, patients in the IV dexmedetomidine group were estimated to have odds of hypotension 2.03 times higher than those in the control group [odds ratio (OR) 1.1-3.74, p = 0.023]. No significant differences existed in postoperative day one hemoglobin between the patients who received IV dexmedetomidine, and those that did not. There was also no difference between the two groups when discharge disposition was evaluated.

**Table 2:** Adjusting for all other variables in the model, patients in the dexmedetomidine group are estimated to have odds of hypotension 2.03 times higher than those in the control group (p = 0.023).

Comparison	Odds Ratio (95% CI)	P-Value
Dex IV: Yes vs. No	2.03 (1.1, 3.74)	0.023
Age (Continuous)	1.01 (0.981, 1.04)	0.5
BMI (Continuous)	1.07 (1.01, 1.13)	0.016
Sex: M vs. F	0.388 (0.205, 0.733)	0.0035
ASA Score: 3 or 4 vs. 1 or 2	1.32 (0.718, 2.43)	0.37
Regional Nerve Block: (Yes-fascia iliaca) vs. No	0.790 (0.3632, 1.72)	0.553
Regional Nerve Block: (Yes-quadratus lumborum or femoral nerve) vs. No	0.223 (0.0418, 1.19)	0.078
Regional Nerve Block: (Yes-quadratus lumborum or femoral nerve) vs. (Yes-fascia iliaca)	0.282 (0.0467, 1.70)	0.167
Pericapsular Injection: Yes vs. No	0.878 (0.34, 2.26)	0.79
Anesthesia Type: Other vs. General	0.673 (0.0811, 5.58)	0.71
Anesthesia Type: Spinal vs. General	1.433 (0.7563, 2.72)	0.27
Anesthesia Type: Spinal vs. Other	2.131 (0.2597, 17.48)	0.48
Approach: Posterior vs. Anterior	0.408 (0.108, 1.54)	0.19
Opioid Use at Baseline: Yes vs. No	1.04 (0.566, 1.92)	0.89

Analysis was then performed for patients who received general and spinal anesthesia. First patients were analyzed who had received general anesthesia, in combination with or without IV dexmedetomidine. Results were tabulated from the linear mixed effects model of the effect dexmedetomidine had on MME at POD 0-3. Patients who underwent general anesthesia and received IV dexmedetomidine exhibited a trend towards increased opioid use; these patients were estimated to have an elevated post-operative MME use of 27% (p= 0.11), 18% (p=0.27), 11% (p=0. 50), and 28% (p= 0.19) compared to than those in the control group at POD 0-3, respectively. Although this observed increase did not reach a level of significance between the two groups, the expected significant reduction in opioid use in the patients who received IV dexmedetomidine was not observed. Patients in the IV dexmedetomidine group who received general anesthesia did not have a significant increase in odds of postoperative hypotension compared to the control, and no significant difference existed in discharge disposition between patients who underwent general anesthesia and received IV dexmedetomidine and those who did not.

Subgroup analysis was then performed in patients who received spinal anesthesia, in combination with or without IV dexmedetomidine. Results were tabulated from the linear mixed effects model of the effect dexmedetomidine and other patient and surgical characteristics had on MME at postoperative days zero through three. When adjusting for all other variables in the model, spinal anesthesia patients who had received IV dexmedetomidine are estimated to have an increased postoperative MME use of 19% (p=0.31), 8% (p = 0.646), 12% (p= 0.556) and 53% (p = 0.073) higher than those in the control group at POD 0-3, respectively. The expected opioid-sparing effect of IV dexmedetomidine was not observed, and although the increased opioid use in the patients who received spinal anesthesia did not reach a level of significance, a trend towards increased opioid use in this group existed on postoperative day three. When adjusted for all other variables in the model, including preoperative opioid use at baseline, the effect IV dexmedetomidine and other patient and surgical characteristics had on hypotension at postoperative day zero is listed in Table 3. Patients in the IV dexmedetomidine group who received spinal anesthesia are estimated to have odds of hypotension 347% greater than those in the control group, who underwent spinal anesthesia without the administration of IV dexmedetomidine (OR 1.43-8.43, p = 0.006).

**Table 3:** Effect of Dexmedetomidine and Patient/Surgical Characteristics on Hypotension (Min MAP < 60) at POD 0, Subjects who Received Spinal Anesthesia. Adjusting for all other variables in the model, patients in the dexmedetomidine group are estimated to have odds of hypotension 3.47 times higher than those in the control group (p = 0.006).

Comparison	Odds Ratio (95% CI)	P-Value
Dex IV: Yes vs. No	3.47 (1.43, 8.43)	0.006
Age (Continuous)	0.987 (0.949, 1.03)	0.53
BMI (Continuous)	1.05 (0.965, 1.14)	0.27
Sex: M vs. F	0.439 (0.183, 1.05)	0.065
ASA Score: 3 or 4 vs. 1 or 2	1.81 (0.742, 4.4)	0.19
Regional Nerve Block: (Yes-fascia iliaca) vs. No	8.59e-01 (0.334, 2.21)	0.75
Regional Nerve Block: (Yes-quadratus lumborum or femoral nerve) vs. No	6.16e-12 (0.000, Inf)	1.00
Regional Nerve Block: (Yes-quadratus lumborum or femoral nerve) vs. (Yes-fascia iliaca)	7.17e-12 (0.000, Inf)	1.00
Pericapsular Injection: Yes vs. No	0.731 (0.297, 1.8)	0.5
Opioid Use at Baseline: Yes vs. No	0.19 (0.0594, 0.605)	0.005

## Discussion

Our study failed to find a reduction in postoperative MME with the use of IV dexmedetomidine. In fact, IV dexmedetomidine use intraoperatively resulted in a trend towards higher MME use at various time points postoperatively. Usualo et al. found a 23.6 mg reduction in cumulative postoperative opioid use in patients who had received 50 mg dosing of IN dexmedetomidine [5]. In our patient population, the reported opioid-sparing benefits of IN dexmedetomidine were not reproduced with the use of IV dexmedetomidine. This finding is not completely surprising as a Cochrane review of postoperative opioid consumption after abdominal surgery in 492 patients showed a modest, but not clinically important difference in postoperative opioid consumption and postoperative pain scores [8]. Patients who received IV dexmedetomidine in our study had a significantly higher postoperative day zero opioid requirement regardless of the type of anesthesia used, general versus spinal. A trend towards higher daily MME on postoperative day three was also observed compared to controls. Patients who received IV dexmedetomidine not only consumed more opioids based on MME equivalents on postoperative day zero, but the concurrent use of IV dexmedetomidine in subjects who also underwent spinal anesthesia led to a 247% higher risk of postoperative hypotension on postoperative day zero compared to controls in the spinal anesthesia group who had not received IV dexmedetomidine (p = 0.006). The lack of observed opioid sparing benefit, in combination with the predilection for precipitating postoperative hypotension in patients who had undergone spinal anesthesia, raises concern about the use of IV dexmedetomidine as an anesthesia adjunct. This is especially true in the setting of ambulatory arthroplasty, where postoperative hypotension is a critical risk factor for failure of same-day discharge [9].

The successful implementation and execution of a same-day arthroplasty program require several critical criteria to be met for the safe discharge of a patient, including the ability to complete a formal physical therapy protocol, obtaining adequate postoperative pain control from oral analgesics, with stable vital signs in the postoperative period [10]. Postoperative hypotension after THA severely impacts a patient’s progress through an ambulatory surgery protocol. In their meta-analysis, Hoffman et al. found that one of the major three reasons for failure to discharge as planned for same-day arthroplasty was postoperative hypotension [11]. In our patient population, an increased odds ratio of postoperative hypotension of 2.47 was observed, when IV dexmedetomidine was used with spinal anesthesia. This raises a concern for same-day discharge failure, in the ambulatory setting, if IV dexmedetomidine is used with spinal anesthesia.

The novelty of this study lies in the fact that the delivery mechanism was via intravenous format, a more commonly used method than intranasal, and the inclusion of patients undergoing THA regardless of anesthesia type, ASA score, or age. To our knowledge, the present study is the largest investigation to report the perioperative effects of IV dexmedetomidine in the setting of primary THA.

However, limitations inherent to a retrospective study exist, the patients were not randomized into treatment groups, and potential confounders did exist between the group of patients who received IV dexmedetomidine and the controls that did not. These possible confounders included: BMI, the use of general anesthesia, surgical approach, regional nerve block, intraoperative opioid administration, opioid use at baseline and gabapentinoid use at baseline, and the selection bias inherent in the choice to administer IV dexmedetomidine. The dose and administration are provider dependent and the addition of IV dexmedetomidine could have been given to patients thought to likely need more analgesia, those who were harder to “sedate”, or those who used more opioids at baseline. Additionally, our multimodal pain management regimen may vary between other institutions, including the use of IV dexamethasone, which can contribute to confounding. We attempted to control for these confounders using the linear mixed effects model and adjusted for all other variables in relation to the dependent variable being analyzed, to minimize the confounding inherent in this retrospective study. The selection of patients to receive IV dexmedetomidine was left at the discretion of the anesthesiologist, which introduces a selection bias that we are unable to control for in the current study model. Furthermore, in anesthetized patients, in general, pain medication administration is often increased when heart rate and blood pressure increase. The hemodynamic effects of dexmedetomidine might confound the pain assessment and as such, be responsible for a reduced intraoperative opioid consumption. The use of a linear mixed effects model including random intercepts for subject and surgeon and fixed effects for dexmedetomidine use, age, sex, BMI, ASA score, type or use of regional nerve block, use of pericapsular injection, type of anesthesia, approach, opioid use at baseline, hospital day, and the interaction of hospital day with all other variables was applied to minimize the confounding between variables, and each variable was investigated independently while taking all other variables in to account to minimize bias in the reported outcomes.

## Conclusions

IV dexmedetomidine should be used cautiously as an anesthesia adjunct in the setting of primary THA, as the experience at our institution showed a 347% increased odds of postoperative hypotension when used with spinal anesthesia, without the observed opioid sparing benefit reported in earlier studies. In the ambulatory arthroplasty setting, postoperative hypotension is a significant barrier to same-day discharge. Further investigation with randomized control trials is warranted to determine the causative nature of IV dexmedetomidine and postoperative hypotension in the primary THA population when using spinal anesthesia. Based upon our analysis we currently have limited its use, especially in the ambulatory setting, until further research elucidates the safety profile of IV dexmedetomidine and its association with postoperative hypotension.
